# The effects of an 8-year individualised lifestyle intervention on food consumption and nutrient intake from childhood to adolescence: the PANIC Study

**DOI:** 10.1017/jns.2022.13

**Published:** 2022-06-02

**Authors:** Taisa Sallinen, Anna Viitasalo, Niina Lintu, Juuso Väistö, Sonja Soininen, Henna Jalkanen, Eero A. Haapala, Santtu Mikkonen, Ursula Schwab, Timo A. Lakka, Aino-Maija Eloranta

**Affiliations:** 1 University of Eastern Finland Library Kuopio, Kuopio, Finland; 2 Institute of Public Health and Clinical Nutrition, School of Medicine, University of Eastern Finland, Kuopio, Finland; 3 Institute of Biomedicine, School of Medicine, University of Eastern Finland, Kuopio, Finland; 4 Social and Health Center, Varkaus, Finland; 5 Faculty of Sport and Health Sciences, University of Jyväskylä, Jyväskylä, Finland; 6 Department of Environmental and Biological Sciences, University of Eastern Finland, Kuopio, Finland; 7 Department of Applied Physics, University of Eastern Finland, Kuopio, Finland; 8 Department of Medicine, Endocrinology and Clinical Nutrition, Kuopio University Hospital, Kuopio, Finland; 9 Department of Clinical Physiology and Nuclear Medicine, School of Medicine, Kuopio University Hospital, University of Eastern Finland, Kuopio, Finland; 10 Kuopio Research Institute of Exercise Medicine, Kuopio, Finland

**Keywords:** Adolescents, Children, Food consumption, Intervention, Nutrient intake, PANIC Study, PANIC, Physical Activity and Nutrition in Children

## Abstract

We aimed to investigate the effects of a long-term, individualised, family-based lifestyle intervention on food consumption and nutrient intake from childhood to adolescence. We conducted an 8-year diet and physical activity intervention study in a population sample of children aged 7–9 years at baseline in 2007–2009. We allocated the participants to the intervention group (*n* 306) and the control group (*n* 198). We assessed diet by 4-d food records at baseline, 2-year follow-up and 8-year follow-up. We analysed the data using linear mixed-effects models adjusted for age at baseline and sex. The consumption of vegetables and vegetable oil-based spreads (fat ≥60 %) increased in the intervention group but did not change in the control group (*P* < 0⋅001 for time×group interaction). The consumption of fruits and berries increased in the intervention group but decreased in the control group (*P* = 0⋅036). The consumption of high-fat cheese (*P* = 0⋅029), butter-based spreads (*P* = 0⋅001) and salty snacks (*P* = 0⋅028) increased less, and the consumption of low-fat cheese (*P* = 0⋅004) increased more in the intervention group than in the control group. Saturated fat intake (*P* = 0⋅001) increased less, and the intakes of dietary fibre (*P* = 0⋅003), vitamin D (*P* = 0⋅042) and vitamin E (*P* = 0⋅027) increased more in the intervention group than in the control group. The intakes of vitamin C (*P* < 0⋅001) and folate (*P* = 0⋅001) increased in the intervention group but decreased in the control group. To conclude, individualised, family-based lifestyle intervention altered food choices towards more recommended diet and resulted in enhanced diet quality from childhood to adolescence.

## Introduction

Lifestyle changes, such as enhancing diet and increasing physical activity, initiated in childhood play a crucial role in preventing overweight, type 2 diabetes and cardiovascular diseases^(1–4)^. Lifestyle interventions have been rather effective in treating overweight and other cardiometabolic risk factors among children in the short term^(5,6)^, but these effects have been modest in the long term^(7,8)^. The reason for the weak long-term effects of these interventions may be that the lifestyle changes have been relatively small or have not been maintained throughout the intervention period among children with these risk factors. Moreover, few long-term lifestyle intervention studies have targeted general populations of children and adolescents with relatively low levels of cardiometabolic risk factors^(9,10)^.

We have previously reported that our individualised and family-based diet and physical activity intervention increased the consumption of vegetables, high-fat vegetable oil-based spreads and low-fat milk and decreased the consumption of butter-based spreads during the first 2 years of intervention^(11)^. Accordingly, the lifestyle intervention increased the plasma proportion of polyunsaturated fatty acids and decreased the proportion of some saturated fatty acids^(12)^, decreased the plasma concentration of low-density lipoprotein cholesterol^(13)^ and attenuated the increase in insulin resistance^(14)^ during the first two intervention years.

We report here the effects of our lifestyle intervention that was less intensive after the 2-year follow-up but was continued with annual counselling sessions until the 8-year follow-up on food consumption and nutrient intake to provide evidence that the beneficial dietary changes adopted in childhood can be maintained until adolescence.

## Methods

### Study design and participants

The Physical Activity and Nutrition in Children (PANIC) Study is a controlled lifestyle intervention study, the aim of which is to investigate the effects of a combined diet and physical activity intervention on cardiometabolic risk factors in a population sample of children from the city of Kuopio, Finland. The Research Ethics Committee of the Hospital District of Northern Savo approved the study protocol in 2006 (Statement 69/2006) and in 2015 (Statement 422/2015). Written informed consent was acquired from the parent or caregiver of each child and every child provided assent to participation. The PANIC Study has been carried out in accordance with the principles of the Declaration of Helsinki as revised in 2008.

**Fig. 1.**
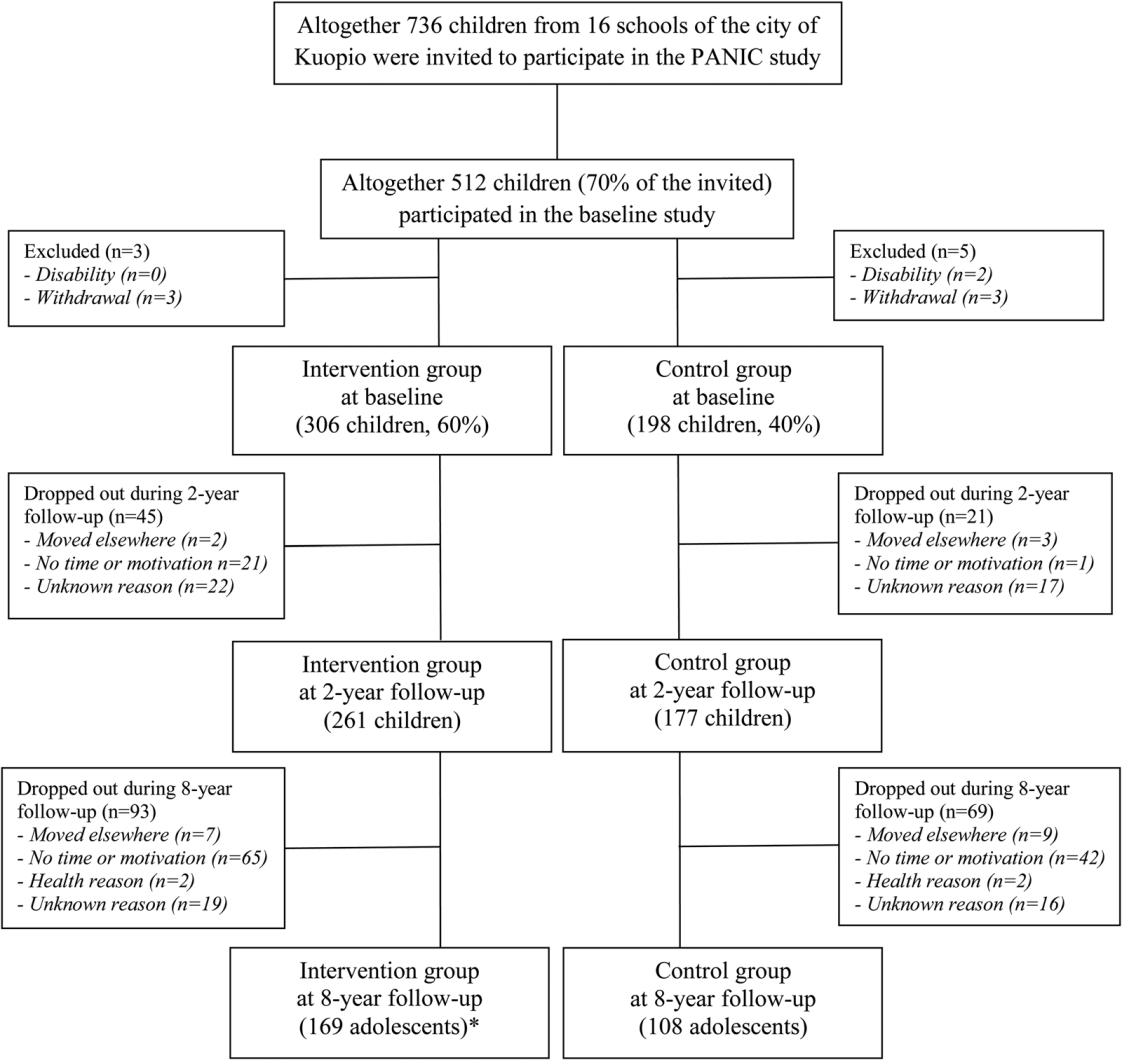
Flowchart of the Physical Activity and Nutrition in Children (PANIC) Study. *One participant who did not attend the 2-year follow-up because of no time or motivation attended the 8-year follow-up measurements.

We invited 736 children 6–9 years of age who started the first grade in 16 primary schools of Kuopio in 2007–2009 (Fig. 1). Altogether 512 children (248 girls, 264 boys), who accounted for 70 % of those invited, participated in the baseline examinations in 2007–2009. The participants did not differ in age, sex or body mass index – standard deviation score (BMI-SDS) from all children who started the first grade in the city of Kuopio in 2007–2009 based on data from the standard school health examinations performed for all Finnish children before the first grade. We excluded six children from the study at baseline because of physical disabilities that could hamper participation in the intervention or no time or motivation to attend the study. We also excluded two children whose parents later withdrew their permission to use the data of their children.

We allocated the children from nine schools to a combined physical activity and dietary intervention group (306 children, 60 %) and the children from seven schools to a control group (198 children, 40 %) to avoid contamination in the control group by any local or national health promotion programmes that could have been initiated in the study region during the follow-up period. We also proportionally matched the intervention and control groups according to the location of the schools (urban *v*. rural) to minimise sociodemographic differences between the groups. We included more children in the intervention group than in the control group because of a larger number of dropouts expected in the intervention group and to retain a sufficient statistical power for comparison between the groups. The children, their parents or people being responsible for the examination visits or the measurements were not blinded to the group assignment.

Of all 504 children who participated in the baseline examinations, 438 (87 %) of them attended the 2-year follow-up examinations, and 277 (55 %) of them attended the 8-year follow-up examinations (Fig. 1). At 2-year follow-up, 261 children were from the intervention group (85 %) and 177 children were from the control group (89 %). At 8-year follow-up, 169 (55 %) adolescents were from the intervention group and 108 (55 %) adolescents were from the control group. Those who participated in the 8-year follow-up examinations did not differ in age (7⋅6 *v*. 7⋅7 years, respectively, *P* = 0⋅173), BMI-SDS (−0⋅2 *v*. −0⋅1, respectively, *P* = 0⋅195) or the distribution of sex (45⋅5 *v*. 51⋅5 % females, respectively, *P* = 0⋅176) or study groups (61⋅0 *v*. 60⋅4 % intervention, *P* = 0⋅880) at baseline from those who dropped out.

The median (interquartile range) of 2-year follow-up time was 2⋅1 (2⋅1–2⋅2) years in both groups. The median (interquartile range) of 8-year follow-up time was 8⋅3 (8⋅1–8⋅3) for the intervention group and 8⋅1 (8⋅0–8⋅3) for the control group. Data on diet were available for 423 children at baseline, for 389 children at 2-year follow-up and for 221 adolescents at 8-year follow-up.

### Diet and physical activity intervention

The goals of the individualised and family-based diet and physical activity intervention were to (1) decrease the consumption of significant sources of saturated fat and particularly high-fat dairy and meat products, (2) increase the consumption of significant sources of unsaturated fat and particularly high-fat vegetable oil-based margarines, vegetable oils and fish, (3) increase the consumption of vegetables, fruits and berries, (4) increase the consumption of significant sources of fibre and particularly whole grain products, (5) decrease the consumption of significant sources of sugar and particularly sugar-sweetened beverages, sugar-sweetened dairy products and candy, (6) decrease the consumption of significant sources of salt and the use of salt in cooking, (7) increase total physical activity by emphasising its diversity, (8) decrease total and particularly screen-based sedentary behaviour and (9) avoid excessive energy intake.

The intervention during the first 2 years included six diet and physical activity counselling sessions consisting of 30–45 min of diet counselling and 30–45 min of physical activity counselling for the children and their parents at the research site. The six counselling sessions occurred 0⋅5, 1⋅5, 3, 6, 12 and 18 months after baseline. In these counselling sessions, the children and their parents received individualised advice from clinical nutritionists and specialists in exercise medicine on how to improve diet, increase physical activity and decrease sedentary behaviour among children in everyday conditions. Each counselling session had a specific topic on diet, physical activity and sedentary behaviour according to the intervention goals and included practical tasks on these topics for the children. The intervention during the first 2 years has been described in more detail previously^(14)^.

After the 2-year follow-up examinations, the diet and physical activity intervention was continued less intensively with one counselling session per year until the 8-year follow-up examinations. The session consisted of 30 min of diet counselling and 30 min of physical activity counselling. The intervention included individualised diet and physical activity counselling sessions occurring 3, 5, 6 and 7 years after baseline at the research site and a group-based counselling session at schools 4 years after baseline. The participants were able to attend the counselling sessions occurring 3, 5, 6 and 7 years after baseline with or without their parents, who were given individualised counselling in a separate room. Each of these counselling sessions had a specific topic on diet, physical activity and sedentary behaviour according to the intervention goals. The group-based counselling session at schools 4 years after baseline included an active lesson with practical tasks.

The children and their parents in the control group received general verbal and written advice on health-improving diet and physical activity at baseline but no active intervention.

### Assessment of diet

We assessed the consumption of food and drinks and the intake of nutrients using food records^(15)^. The food records covered four predefined and consecutive days, including two weekdays and two weekend days or three weekdays and one weekend day. Two food records (0⋅5 %) at 2-year follow-up covered 3 d and consisted of two weekdays and one weekend day and were also included in the analyses. The clinical nutritionists, which were trained based on the protocol of the study, were in charge of giving the instructions about the food records to the study participants at the research site during the study visits. At baseline and 2-year follow-up, a clinical nutritionist instructed the parents to record all food and drinks consumed by their child using household or other measures, such as tablespoons, decilitres and centimetres. At 8-year follow-up, the adolescents were instructed to record their food and drink consumption by themselves. A clinical nutritionist checked the returned food records together with the children and their parents at baseline and 2-year follow-up and with the adolescents at 8-year follow-up and filled in any missing information. We calculated food consumption and nutrient intake using the Micro Nutrica® dietary analysis software, Version 2.5. The software is based on detailed information about the nutrient content of foods in Finland and other countries^(16)^. Moreover, a clinical nutritionist updated the software by adding new food items and products with their actual nutrient content based on new data in the Finnish food composition database^(17)^ or received from the producers. The use of vitamin and mineral supplements was not included in these analyses.

### Assessment of body size

Body height and weight were assessed by trained professionals in the morning at the research site after the participants had fasted for 12 h. Body height was assessed using a wall-mounted stadiometer and body weight using the InBody^®^ 720 bioelectrical impedance device (Biospace, Seoul, Korea), with the weight assessment integrated into the system. We computed age- and sex-standardised BMI-SDS using Finnish references^(18)^. Overweight and obesity were defined using the International Obesity Task Force criteria, corresponding to an adult BMI cut-point at 25 for overweight and at 30 for obesity^(19)^.

### Statistical analyses

Statistical analyses were performed using the IBM SPSS Statistics®, Version 27 (IBM Corp., Armonk, NY, USA). *P* values of <0⋅05 were used to indicate statistical significance, based on two-sided testing. Sample size calculations have been explained in detail in Lakka *et al.*^(14)^. We compared baseline characteristics between the intervention and control groups by linear mixed-effects models with cluster-robust standard errors, except body weight status for which comparison was performed by generalised linear mixed-effects models with an ordered structure to account for the clustering effect of schools.

We studied the effects of the intervention on food consumption and nutrient intake using linear mixed-effects models with repeated outcome measures (baseline, 2-year follow-up and 8-year follow-up) with children considered as subjects in the mixed model structure. We used a model adjusted for age at baseline and sex, including main effects for time and time-by-study group interaction as follows: OUTCOME_it_ = (*β*_0_ *+ u_i_*) *+ β*_1_age + *β*_2_sex + (*β*_3_ + *v_i_*)time + *β*_4_study group *×* time *+ ɛ*_it_. In this model, OUTCOME_it_ are observations for subject *i* at baseline, 2-year follow-up and 8-year follow-up; *β*_0_ is the intercept; *β*_1_, *β*_2_, *β*_3_ and *β*_4_ are the regression coefficients for age, sex, time and study group × time, respectively; *u_i_* are random, subject-specific intercepts; *v_i_* are corresponding random slopes for follow-up time and ɛ*_it_* is the error for subject *i* at time *t*.

We used the Bayesian information criterion as a measure of model adequacy, a lower value indicating a better model with optimal balance between complexity and good fit. We *a priori* decided to choose the model with the lowest value of the Bayesian information criterion as the final model for each variable. We fitted all possible models by allowing or ignoring possible clustering on the subject level for each dependent variable. The data for food consumption and nutrient intake showed the best fit with the model in which a random intercept or a random intercept and a random regression coefficient of gender were modelled on the subject level by using an independent variance structure.

One of the typical problems related to using the time-by-study group interaction is the phenomenon of regression to the mean due to the differences between the intervention and control groups at baseline^(20)^. There were no differences in food consumption or nutrient intake between the study groups at baseline, except for the consumption of high-fat (≥1 %) milk (*P* = 0⋅019) and low-fat (<1 %) milk (*P* = 0⋅035) and the intake of vitamin C (*P* = 0⋅021). Therefore, we did not include the study group in the model, except for these variables, to allow for the regression to the mean phenomenon. Instead, baseline values in the study groups are reflected in the intercept of the model.

## Results

### Baseline characteristics

There were no differences in the characteristics of children between the intervention and control groups at baseline (Table 1).

**Table 1.** Characteristics of children at baseline

	Intervention	Control	*P*
Number	306	198	
Age, years	7⋅6 (0⋅4)	7⋅6 (0⋅4)	0⋅99
Sex, *n* (%)			0⋅52
Girls	144 (47⋅1)	99 (50⋅0)	
Boys	162 (52⋅9)	99 (50⋅0)	
Body height, cm	129 (5⋅5)	129 (5⋅9)	0⋅85
Body weight, kg	27⋅0 (4⋅8)	26⋅8 (5⋅3)	0⋅78
Body mass index- standard deviation score	−0⋅2 (1⋅1)	−0⋅2 (1⋅1)	0⋅66
Body weight status, *n* (%)			0⋅75
Normal weight	267 (86⋅3)	173 (87⋅4)	
Overweight	30 (9⋅8)	15 (7⋅6)	
Obesity	12 (3⋅9)	10 (5⋅1)	

The values are means (standard deviation) for continuous variables and *n* (%) for categorical variables. *P* values are shown for differences between the intervention and control groups from linear mixed-effects models with cluster-robust standard errors, except that numbers (percentages) for body weight status and *P* values for their differences between the intervention and control groups are from eneralized linear mixed-effects models with an ordered structure to account for the clustering effect of schools.

### Effects of 8-year intervention on food consumption

The consumption of vegetables increased in the intervention group but did not change in the control group over 8 years of intervention (Table 2). The consumption of fruits and berries increased in the intervention group but decreased in the control group. The consumption of high-fat (>17 %) cheese increased less, and the consumption of low-fat (≤17 %) cheese increased more in the intervention group than in the control group. The consumption of butter and butter-based spreads increased less in the intervention group than in the control group. The consumption of vegetable oil-based spreads (fat ≥60 %) increased in the intervention group but did not change in the control group. The consumption of salty snacks increased less in the intervention group than in the control group.

**Table 2.** Mean (95 % confidence interval) food consumption in the intervention and control groups at the baseline, 2-year follow-up and 8-year follow-up study

	Baseline		2-year follow-up		8-year follow-up		*P* for time^a^ group interaction
	Intervention	Control	Intervention	Control	Intervention	Control	
High-fibre (≥5 %) products (g/d)^a^	62⋅7 (56⋅9−68⋅5)	62⋅1 (55⋅0−69⋅3)	75⋅9 (69⋅8−82⋅0)	73⋅2 (65⋅8−80⋅6)	90⋅4 (82⋅7−98⋅1)	78⋅2 (68⋅2−88⋅3)	0⋅30
Low-fibre (<5 %) products (g/d)^b^	112 (105−120)	116 (107−125)	109 (101−116)	104 (94⋅8−113⋅8)	113 (103−123)	124 (111−137)	0⋅42
Potatoes (g/d)^a^	76⋅5 (70⋅3−82⋅7)	76⋅5 (68⋅8−84⋅2)	75⋅1 (68⋅6−81⋅6)	75⋅2 (67⋅3−83⋅1)	85⋅3 (76⋅9−93⋅6)	77⋅1 (66⋅3−88⋅0)	0⋅70
Vegetables (g/d)^a^	97⋅1 (89⋅2−105)	111 (101−120)	109 (101−117)	97⋅8 (87⋅9−108)	133 (123−144)	111 (97⋅3−124)	0⋅001
Fruits and berries (g/d)^a^	107 (95⋅9−118)	110 (96⋅5−123)	108 (97⋅0−120)	93⋅3 (79⋅5−107)	113 (98⋅3−127)	83⋅2 (64⋅3−102)	0⋅04
High-fat (≥1 %) milk (g/d)^a^^,^^c^	171 (146−195)	224 (194−254)	97⋅0 (71⋅7−122)	186 (156−217)	75⋅9 (44⋅6−107)	120 (79⋅9−161)	0⋅17
Low-fat (<1 %) milk (g/d)^b^^,^^c^	404 (368−439)	339 (295−382)	473 (436−510)	343 (298−387)	366 (321−412)	260 (201−318)	0⋅13
High-fat (≥1 %) sour milk products (g/d)^b^	89⋅5 (80⋅2−98⋅9)	77⋅3 (65⋅8−88⋅8)	66⋅6 (56⋅8−76⋅4)	65⋅8 (53⋅9−77⋅6)	55⋅4 (42⋅9−67⋅9)	48⋅8 (32⋅6−65⋅0)	0⋅41
Low-fat (<1 %) sour milk products (g/d)^b^	18⋅7 (12⋅1−25⋅4)	18⋅5 (10⋅3−26⋅7)	19⋅4 (12⋅4−26⋅4)	17⋅5 (9⋅0−25⋅9)	30⋅4 (21⋅5−39⋅3)	14⋅8 (3⋅3−26⋅3)	0⋅21
High-fat cheese (>17 %) (g/d)^b^	7⋅5 (6⋅0−9⋅0)	9⋅1 (7⋅3−10⋅9)	6⋅9 (5⋅3−8⋅4)	9⋅8 (7⋅9−11⋅7)	15⋅2 (13⋅2−17⋅2)	18⋅3 (15⋅7−20⋅9)	0⋅03
Low-fat cheese (≤17 %) (g/d)^a^	7⋅1 (5⋅4−8⋅8)	7⋅9 (5⋅8−10⋅1)	6⋅7 (7⋅9−11⋅5)	7⋅2 (5⋅0−9⋅4)	15⋅2 (12⋅8−17⋅5)	8⋅9 (5⋅9−11⋅9)	0⋅004
Ice cream and pudding (g/d)^a^	24⋅8 (21⋅3−28⋅3)	30⋅3 (26⋅0−34⋅6)	23⋅4 (19⋅7−27⋅0)	24⋅6 (20⋅2−29⋅0)	9⋅5 (4⋅8−14⋅2)	16⋅2 (10⋅0−22⋅3)	0⋅09
Fish (g/d)^a^	15⋅5 (12⋅6−18⋅3)	16⋅3 (12⋅7−19⋅8)	18⋅2 (15⋅2−21⋅2)	16⋅8 (13⋅2−20⋅4)	18⋅1 (14⋅3−22⋅0)	23⋅4 (18⋅4−28⋅3)	0⋅35
Red meat (g/d)^b^	55⋅6 (50⋅9−60⋅2)	58⋅6 (52⋅9−64⋅3)	59⋅4 (54⋅6−64⋅2)	63⋅1 (57⋅3−69⋅0)	66⋅7 (60⋅5−73⋅0)	65⋅0 (57⋅0−73⋅1)	0⋅64
Sausages (g/d)^b^	22⋅0 (18⋅7−25⋅3)	22⋅1 (18⋅0−26⋅2)	21⋅3 (17⋅9−24⋅8)	21⋅1 (16⋅9−25⋅3)	26⋅9 (22⋅4−31⋅3)	21⋅1 (15⋅4−26⋅9)	0⋅49
Poultry (g/d)^a^	16⋅6 (13⋅1−20⋅0)	18⋅0 (13⋅7−22⋅2)	22⋅3 (18⋅7−25⋅9)	20⋅0 (15⋅7−24⋅4)	39⋅1 (34⋅4−43⋅7)	38⋅1 (32⋅0−44⋅1)	0⋅80
Butter and butter-based spreads (g/d)^b^	5⋅8 (4⋅8−6⋅8)	6⋅1 (4⋅9−7⋅3)	4⋅8 (3⋅7−5⋅8)	8⋅0 (6⋅7−9⋅2)	7⋅6 (6⋅2−8⋅9)	9⋅3 (7⋅6−11⋅1)	0⋅001
Vegetable oil-based spreads (fat 60–80 %) (g/d)^b^	6⋅8 (5⋅4−8⋅2)	7⋅8 (6⋅1−9⋅6)	17⋅0 (15⋅5−18⋅5)	10⋅4 (8⋅6−12⋅2)	12⋅7 (10⋅9−14⋅6)	7⋅3 (4⋅8−9⋅7)	<0⋅001
Vegetable oil-based spreads (fat <60 %) (g/d)^b^	3⋅9 (3⋅1−4⋅7)	3⋅7 (2⋅8−4⋅7)	1⋅7 (0⋅9−2⋅5)	3⋅0 (2⋅0−3⋅9)	0⋅4 (−0⋅6−1⋅5)	2⋅1 (0⋅7−3⋅4)	0⋅06
Vegetable oils (g/d)^b^	4⋅2 (3⋅6−4⋅8)	3⋅9 (3⋅2−4⋅6)	4⋅4 (3⋅8−5⋅0)	4⋅4 (3⋅6−5⋅1)	5⋅8 (5⋅0−6⋅5)	5⋅6 (4⋅6−6⋅6)	0⋅91
Sugar-sweetened beverages (g/d)^b^	128 (110−145)	149 (128−171)	139 (120−157)	161 (139−184)	135 (116−163)	171 (140−202)	0⋅12
Fruit juices (g/d)^b^	32⋅3 (23⋅6−41⋅1)	40⋅3 (29⋅6−51⋅0)	36⋅0 (26⋅9−45⋅1)	44⋅1 (33⋅1−55⋅1)	47⋅6 (36⋅1−59⋅1)	38⋅8 (24⋅0−53⋅7)	0⋅30
Sugar and honey (g/d)^a^	9⋅7 (8⋅6 −10⋅7)	9⋅9 (8⋅7−11⋅2)	6⋅6 (5⋅6−7⋅7)	7⋅0 (5⋅7−8⋅2)	8⋅3 (7⋅0−9⋅7)	8⋅4 (6⋅7−10⋅2)	0⋅97
Candy (g/d)^a^	21⋅2 (17⋅9−24⋅5)	20⋅6 (16⋅5−24⋅6)	24⋅6 (21⋅2−28⋅0)	23⋅9 (19⋅8−28⋅1)	21⋅6 (17⋅3−26⋅0)	18⋅1 (12⋅5−23⋅7)	0⋅81
Chocolate and hot chocolate powder (g/d)^a^	9⋅9 (8⋅1−11⋅6)	9⋅2 (7⋅0−11⋅3)	11⋅0 (9⋅1−12⋅8)	11⋅3 (9⋅1−13⋅6)	11⋅2 (8⋅8−13⋅6)	11⋅6 (8⋅5−14⋅6)	0⋅94
Salty snacks (g/d)^a^	4⋅0 (2⋅3−5⋅8)	4⋅4 (2⋅3−6⋅6)	6⋅7 (4⋅9−8⋅6)	7⋅2 (5⋅0−9⋅4)	6⋅0 (3⋅7−8⋅4)	12⋅0 (8⋅9−15⋅0)	0⋅03

a Only random intercept included in the linear mixed-effects model.

b Random intercept and gender included in the linear mixed-effects model.

c Ths study group included in the model because of baseline differences between the intervention and control groups.

### Effects of 8-year intervention on nutrient intake

The intake of saturated fat increased less, and the intake of dietary fibre increased more in the intervention group than in the control group over 8 years of intervention (Table 3). The intake of vitamin C increased in the intervention group but decreased in the control group. The intakes of vitamin D and vitamin E increased more in the intervention group than in the control group. The intake of folate increased in the intervention group but decreased in the control group.

**Table 3.** Mean (95 % confidence interval) nutrient intake in the intervention and control groups at the baseline, 2-year follow-up and 8-year follow-up study

	Baseline		2-year follow-up		8-year follow-up		*P* for time^a^group interaction
	Intervention	Control	Intervention	Control	Intervention	Control	
Energy (kcal)^a^	1620 (1576−1665)	1672 (1617−1727)	1693 (1647−1740)	1682 (1625−1738)	1862 (1803−1922)	1812 (1735−1889)	0⋅30
Total fat (% of energy intake)^a^	29⋅7 (29⋅0−30⋅4)	30⋅4 (29⋅6−31⋅3)	31⋅0 (30⋅3−31⋅7)	32⋅0 (31⋅2−32⋅9)	34⋅0 (33⋅1−34⋅9)	34⋅7 (33⋅6−35⋅9)	0⋅17
SFA (% of energy intake)^a^	12⋅0 (11⋅7−12⋅3)	12⋅4 (12⋅0−12⋅8)	11⋅6 (11⋅3−12⋅0)	12⋅6 (12⋅2−13⋅0)	12⋅4 (12⋅0−12⋅8)	13⋅1 (12⋅5−13⋅6)	0⋅001
MUFA (% of energy intake)^a^	9⋅9 (9⋅6−10⋅2)	10⋅1 (9⋅7−10⋅4)	10⋅9 (10⋅6−11⋅2)	10⋅9 (10⋅6−11⋅3)	12⋅1 (11⋅8−12⋅5)	11⋅5 (11⋅0−12⋅0)	0⋅21
PUFA (% of energy intake)^a^	4⋅9 (4⋅7−5⋅1)	4⋅9 (4⋅7−5⋅1)	5⋅8 (5⋅6−6⋅0)	5⋅6 (5⋅3−5⋅9)	6⋅3 (6⋅0−6⋅5)	6⋅6 (6⋅2−6⋅9)	0⋅25
Carbohydrates (% of energy intake)^a^	52⋅1 (51⋅4−52⋅8)	51⋅5 (50⋅7−52⋅4)	50⋅7 (50⋅0−51⋅4)	50⋅0 (49⋅1−50⋅8)	47⋅1 (46⋅2−48⋅0)	46⋅6 (45⋅4−47⋅8)	0⋅49
Sucrose (% of energy intake)^a^	12⋅7 (12⋅2−13⋅2)	12⋅7 (12⋅1−13⋅3)	11⋅3 (10⋅7−11⋅8)	11⋅8 (11⋅1−12⋅4)	10⋅4 (9⋅7−11⋅0)	10⋅4 (9⋅6−11⋅3)	0⋅65
Protein (% of energy intake)^a^	16⋅8 (16⋅5−17⋅2)	16⋅7 (16⋅3−17⋅1)	16⋅9 (16⋅5−17⋅3)	16⋅6 (16⋅2−17⋅1)	17⋅5 (17⋅1−18⋅0)	17⋅3 (16⋅7−17⋅9)	0⋅78
Fibre (g/d)^a^	14⋅4 (13⋅8−15⋅0)	14⋅4 (13⋅7−15⋅1)	15⋅7 (15⋅0−16⋅3)	14⋅3 (13⋅5−15⋅0)	17⋅6 (16⋅8−18⋅4)	15⋅7 (14⋅7−16⋅8)	0⋅003
Vitamin C (mg/d)^a^^,^^b^	81⋅7 (76⋅0−87⋅4)	93⋅3 (86⋅3−100⋅3)	87⋅1 (81⋅1−93⋅1)	85⋅4 (78⋅2−92⋅6)	90⋅5 (82⋅9−98⋅2)	72⋅7 (62⋅7−82⋅6)	<0⋅001
Vitamin D (μg/d)^c^	5⋅8 (5⋅4−6⋅2)	6⋅0 (5⋅4−6⋅5)	8⋅4 (8⋅0−8⋅9)	7⋅8 (7⋅3−8⋅4)	10⋅3 (9⋅7−10⋅8)	9⋅1 (8⋅4−9⋅9)	0⋅04
Vitamin E (mg/d)^a^	6⋅6 (6⋅3−7⋅0)	7⋅0 (6⋅6−7⋅4)	8⋅1 (7⋅7−8⋅4)	7⋅5 (7⋅0−7⋅9)	9⋅6 (9⋅1−10⋅0)	9⋅0 (8⋅4−9⋅5)	0⋅03
Folate (μg/d)^a^	188 (181−196)	194 (185−203)	190 (183−198)	185 (176−194)	220 (211−230)	191 (178−203)	0⋅001
Sodium (mg/d)^c^	2405 (2325−2484)	2441 (2343−2539)	2521 (2438−2604)	2566 (2465−2667)	2860 (2754−2966)	2821 (2684−2958)	0⋅79
Calcium (mg/d)^c^	1167 (1119−1215)	1168 (1109−1227)	1183 (1133−1233)	1127 (1067−1187)	1141 (1079−1203)	1034 (954−1114)	0⋅12
Iron (mg/d)^c^	8⋅2 (7⋅9−8⋅5)	8⋅4 (8⋅0−8⋅8)	8⋅5 (8⋅2−8⋅8)	8⋅3 (7⋅9−8⋅6)	9⋅7 (9⋅3−10⋅1)	9⋅1 (8⋅6−9⋅6)	0⋅15

a Only random intercept included in the linear mixed-effects model.

b The study group included in the model because of baseline differences between the intervention and control groups.

c Random intercept and gender included in the linear mixed-effects model.

## Discussion

This 8-year controlled lifestyle intervention study demonstrated that the individualised and family-based diet and physical activity intervention improved diet quality from childhood to adolescence at 15–17 years of age. The consumption of vegetables, fruits and berries, low-fat cheese and vegetable oil-based spreads, as well as the intakes of dietary fibre, vitamin C, vitamin D, vitamin E and folate, increased in the intervention group. Moreover, the consumption of high-fat cheese, butter and butter-based spreads, and salty snacks and the intake of saturated fat increased less in the intervention group than in the control group. The intervention effects on vegetables, vegetable oil-based spreads, butter and butter-based spreads, dietary fibre, vitamin C and vitamin E were observed already at the 2-year follow-up^(11)^, but the other effects were detected only at the 8-year follow-up.

At 2-year follow-up, our intervention increased the consumption of vegetables likely resulting in the increased intake of dietary fibre and vitamin C^(11)^. The results of the present study suggest that in addition to maintaining the increased consumption of vegetables, the participants in the intervention group also increased the consumption of fruits and berries by adolescence. These dietary changes probably led to the maintenance of the increased intake of dietary fibre and vitamin C but also to the increased intake of folate by adolescence. Another family-based dietary intervention initiated in childhood was also found to increase the consumption of vegetables, fruits and berries among adolescents^(21)^. Indeed, parental modelling, encouragement and availability at home have been demonstrated to be important determinants for the consumption of vegetables and fruits among adolescents^(22)^, emphasising family involvement in the interventions targeting increasing the consumption of vegetables, fruits and berries in this age group. Because a diet high in vegetables and fruits in childhood and adolescence has been associated with increased elasticity of arteries in adulthood^(23)^, family-based diet interventions could improve cardiovascular health from adolescence to adulthood.

During the first 2 years, our lifestyle intervention decreased the consumption of high-fat milk and butter-based spreads but increased the consumption of low-fat milk and high-fat vegetable oil products^(11)^. These findings were further supported by a smaller decrease in the plasma proportion of total polyunsaturated fatty acids and linoleic acid and an increase in the plasma proportion of α-linolenic acid in the intervention group than in the control group over 2 years^(12)^. We now observed that the decreased consumption of butter and butter-based spreads and the increased consumption of high-fat vegetable oil-based spreads had been maintained until adolescence in the intervention group. Moreover, we found that the consumption of low-fat cheese increased more, and the consumption of high-fat cheese increased less in the intervention group than in the control group. These improvements in the diet likely resulted in a smaller increase in the intake of saturated fat in the intervention group than in the control group for 8 years. Moreover, the increased consumption of low-fat milk and high-fat vegetable oil-based spreads, both commonly fortified by vitamin D in Finland^(24)^ and being the main sources of vitamin D among Finnish children^(25)^, likely resulted in the increased intake of vitamin D. The increased consumption of high-fat vegetable oil-based spreads, together with the increased consumption of vegetables and fruits, also likely contributed to the increased intake of vitamin E, as they have been reported to be important sources of vitamin E among children^(15)^. In one earlier diet intervention study among children, the proportion of high-fat dairy products of all dairy products decreased from 88 to 14 % in response to parental dietary counselling, and this beneficial effect also resulted in the decreased intake of saturated fat^(26)^. Other previous family-based dietary interventions have also shown a decrease in the intake of saturated fat among children^(10,27)^ and adolescents^(21)^. All these findings together show the beneficial effect of family-based dietary counselling on the quality of dietary fat. These observations are of potential clinical importance, since replacing saturated fat with polyunsaturated fat has been shown to decrease the plasma concentration of low-density lipoprotein cholesterol and to reduce the risk of coronary heart disease in adults^(28)^. We have earlier found that the diet and physical activity intervention decreased plasma levels of low-density lipoprotein cholesterol over 2 years in the present study sample^(13)^. These observations together suggest that the improved quality of dietary fat since childhood may help prevent cardiovascular diseases in adulthood.

We found no effects of the lifestyle intervention on the consumption of foods containing lots of sugar, such as sugar-sweetened beverages and candies, or the intake of sucrose between the intervention and control groups. Another previous study showed a beneficial effect of diet intervention on sucrose intake during the 20-year follow-up from infancy to early adulthood^(21)^. However, similarly to the present study, the intervention effect was evident only in early adulthood but not in adolescence. One explanation for these observations may be that during teen years, the consumption of sugar-sweetened snacks is more strongly influenced by the peers^(29)^ and the school environment^(30)^ than the parents. Thus, the beneficial effects of individualised and family-based counselling on sucrose intake may occur later in life. It is also possible that a decrease in sucrose intake among adolescents may require school-based environmental interventions in addition to individualised and family-based counselling.

A previous study among adolescents with elevated blood pressure reported no effect of a dietary intervention on sodium intake over 3 years^(31)^. Moreover, sodium intake was even higher in the diet intervention group than in the control group in the early adulthood in a diet intervention study after 20-year follow-up from infancy to early adulthood^(21)^. In contrast, we found that the consumption of salty snacks increased less in the intervention group than in the control group over 8 years. However, we observed no intervention effect on total sodium intake. These results may suggest that it is easier to avoid the excessive consumption of foods containing lots of salt, such as salty snacks, than to limit the use of salt in cooking and to choose low-salt options in more frequently consumed products, such as bread and ready meals. Because a high salt intake is known to increase cardiovascular morbidity and mortality among general populations of adults^(32)^, it is evident that more effective interventions to decrease the consumption of salt at young age are needed.

Major strengths of the present study are the long duration from childhood to adolescence, the relatively large population sample examined and the use of individually instructed and reviewed food records to assess food consumption and nutrient intake, the foods and beverages is being advised to be recorded at the time of consumption to minimise the reliance on memory. Moreover, the beneficial effects of the intervention were found in adolescence that is a challenging period because the food choices of adolescents affect their diet more than those of children. However, the consumption of foods and beverages were reported by the parents on behalf of their children at baseline and at the 2-year follow-up but by the adolescents themselves at the 8-year follow-up, and the reports of parents and adolescents may not be completely comparable. Moreover, subjective reporting is always prone to bias, since the participants may alter their diet intentionally or unintentionally during the recording process. Adolescents in the intervention group may have been more likely to be aware of the recommended diet and misreport their diet towards these recommendations than those in the control group. However, the improved plasma fatty acid profile^(12)^ and the decreased plasma levels of low-density lipoprotein cholesterol^(13)^ after 2 years supported the improved diet quality assessed by food records in the intervention group. One also has to be aware that the season when the food record is filled may affect the dietary data. Moreover, filling in the food record is quite laborious to the study participants. To increase the motivation of filling in the food record completely, we gave a calculation about nutrient intakes of one's diet to all the study participants after the study visits at all time points of the PANIC Study.

In conclusion, we found that the 8-year individualised and family-based diet and physical activity improved diet quality by increasing the consumption of vegetables, fruits and berries, as well as by replacing the consumption of foods high in saturated fat with the consumption of foods high in unsaturated fat from childhood to adolescence. These changes in response to the lifestyle intervention attenuated the increase in the intake of saturated fat and increased the intake of dietary fibre, vitamin C, folate, vitamin D and vitamin E. Further research is warranted to study whether the improved diet quality can reduce cardiometabolic risk between childhood and adolescence and whether the improved dietary behaviour is maintained until adulthood. Moreover, more effective interventions to decrease salt and sugar intakes among children and adolescents are needed.
